# A Pediatric Case of Autism Spectrum Disorder With a Prostatic Abscess

**DOI:** 10.7759/cureus.26941

**Published:** 2022-07-17

**Authors:** Satoko Takahashi, Tatsuo Fuchigami, Takeshi Furuya, Waka Mizukoshi, Ichiro Morioka

**Affiliations:** 1 Department of Pediatrics, IMS Fujimi General Hospital, Fujimi, JPN; 2 Department of Pediatrics and Child Health, Nihon University School of Medicine, Tokyo, JPN; 3 Department of Pediatric Surgery, IMS Fujimi General Hospital, Fujimi, JPN; 4 Department of Pediatric Surgery, Nihon University School of Medicine, Tokyo, JPN; 5 Department of Radiology, IMS Fujimi General Hospital, Fujimi, JPN

**Keywords:** biased toward nutrition, autistic spectrum disorder, pseudomonas aeruginosa, pediatric patient, prostatic abscess

## Abstract

There are few reports of prostatic and periprostatic abscesses in children, and diagnosis is often difficult due to the lack of early symptoms. In addition, children with autism spectrum disorder may have difficulty reporting symptoms, with and without cognitive impairments. This article reports the case of a five-year-old boy with autism spectrum disorder and multiple prostatic abscesses caused by *Pseudomonas aeruginosa*. He also had various vitamin and mineral deficiencies, presumably related to an unbalanced diet. The patient was treated with antibiotics, vitamins, and trace elements. After his blood vitamin and trace element levels returned to normal, he experienced no fever or relapse. The cause of this prostatic abscess was suggested to involve vitamin and trace element deficiencies.

## Introduction

There are few reports of prostatic and periprostatic abscesses in children, and diagnosis is often difficult because of the lack of early symptoms. In addition, children with autism spectrum disorder (ASD) may have difficulty reporting symptoms, with and without cognitive impairment. We herein report the case of a five-year-old boy with ASD with prolonged intermittent fever and multiple prostatic abscesses.

## Case presentation

The patient was a five-year-old boy with intermittent fever and a body temperature of 38°C that had persisted for one month, pain during urination lasting two weeks, and penile swelling lasting five days before visiting our hospital. He was admitted to our department because his symptoms did not improve despite antibiotic therapy administered by his local doctor. The child had a history of ASD with intellectual disability (intelligence quotient: 27). His picky eating worsened a few months before admission; he only ate polished white rice, “natto,” and vinegared Japanese seaweed “mozuku.”

**Figure 1:**
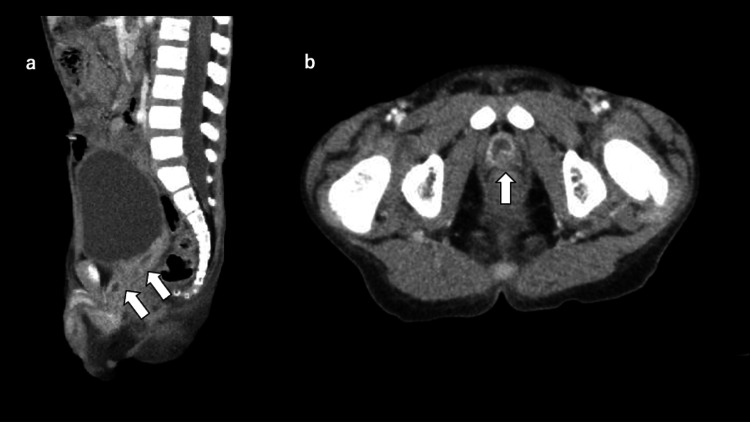
Contrast-enhanced computed tomography A contrast-enhanced computed tomography scan revealed multiple prostatic abscesses with rim enhancing and fluid collection. (a) Sagittal view, (b) axial view.

The patient was 105.0 cm (standard deviation (SD)-1.4) tall and weighed 15 kg (SD-1.5) on admission. The patient’s body temperature was maintained at 40°C, and his penis was reddish and swollen upon admission. His blood examination revealed an elevated white blood cell count (20, 900/μL) with a neutrophil predominance (76.9%) and elevated blood urea nitrogen (15.0 mg/dL). The patient’s C-reactive protein level was 0.24 mg/dL. Catheter urinalysis showed an elevated white blood cell count of 100 /high power field, and urine culture showed *Pseudomonas aeruginosa* (*P. aeruginosa*) at 104 colony-forming units/mL. Contrast-enhanced computed tomography was performed, which revealed areas of contrasted margins and interior water density in the cephalad and apex areas of the prostate (Figure 1).

Intravenous cefotaxime administration (100 mg/kg/day) was initiated with the diagnosis of multiple prostatic abscesses. The patient’s fever quickly resolved and blood test results improved. Antibiotic therapy was changed to oral tosufloxacin (TFLX) (180 mg/day), and the patient was discharged on the sixth day of hospitalization.

However, the patient developed fever again 20 days after hospital discharge and 10 days after TFLX treatment ended; he was admitted for the second time. Upon hospital admission, a urine culture test was performed for urinary tract infection (UTI) and prostate gland infection, revealing *P. aeruginosa*. Intravenous administration of ceftazidime (90 mg/kg/day) was initiated. His fever decreased, and a normal body temperature was maintained. His mother believed that the patient had difficulty seeing, and he could not open his eyes, so brain computed tomography and magnetic resonance imaging (MRI) scans were performed; however, no abnormalities were found.

Due to the child’s poor eating habits, a blood test including serum vitamin and trace element levels was performed. Decreased levels of retinol-binding protein (0.4 mg/dL), vitamin C (＜0.2 μg/mL), total carnitine (42.8 μmol/L), and zinc (53 μg/dL) levels were observed. The patient’s vitamin B1 (32 pg/mL), vitamin B2 (93.7 ng/mL), vitamin B12 (453 pg/mL), 25-hydroxyvitamin D (21.7 ng/mL), folic acid (15.9 ng/mL), and copper (94 μg/dL) were within the normal range. In the immunological examination, immunoglobulin G (1666 mg/dL), immunoglobulin A (312 mg/dL), immunoglobulin M (128 mg/dL), complement 3 (151.8 mg/dL), complement 4 (26.9 mg/dL), and 50% hemolytic unit of complement (47.3 U/mL) levels were normal (Table 1).

**Table 1:** Laboratory data (serum vitamin, trace element, and immunological data) RBP: retinol-binding protein; 25-(OH)D: 25-hydroxyvitamin D; Zn: zinc; Cu: copper; IgG: immunoglobulin G; IgA: immunoglobulin A; IgM: immunoglobulin M; C3: complement 3; C4: complement 4; CH50: 50% hemolytic unit of complement

Test	Result	Reference values	Units
RBP	0.4	2.7-6.0	mg/dL
Vitamin B1	32	24-66	pg/mL
Vitamin B2	93.7	66.1-111.4	ng/mL
Vitamin B12	453	233-914	pg/mL
Vitamin C	<0.2	5.5-16.8	μg/mL
25-(OH)D	21.7	10-30	ng/mL
Folic acid	15.9	2.4-10.0	ng/mL
Zn	53	80-130	μg/dL
Total carnitine	42.8	45-91	μmol/L
Cu	94	70-132	μg/dL
IgG	1666	605-1460	mg/dL
IgA	312	36-221	mg/dL
IgM	128	80-322	mg/dL
C3	151.8	84-151	mg/dL
C4	26.9	17-40	mg/dL
CH50	47.3	25-48	U/mL

Together with the clinical symptoms and vitamin A and vitamin C deficiencies, the oral administration of multivitamins consisting of vitamins A, C, B1, and folic acid was started. Contrast-enhanced abdominal computed tomography was performed. As in the previous case, the prostate’s margins on the cephalic side and near the prostate’s apex were contrast-enhanced, and the interior showed water density. The area was slightly smaller than the previous area, and no new lesions were observed. On the seventh day of hospitalization, the patient showed a tendency toward fever resolution and was discharged on the ninth day after switching to oral TFLX therapy.

**Figure 2:**
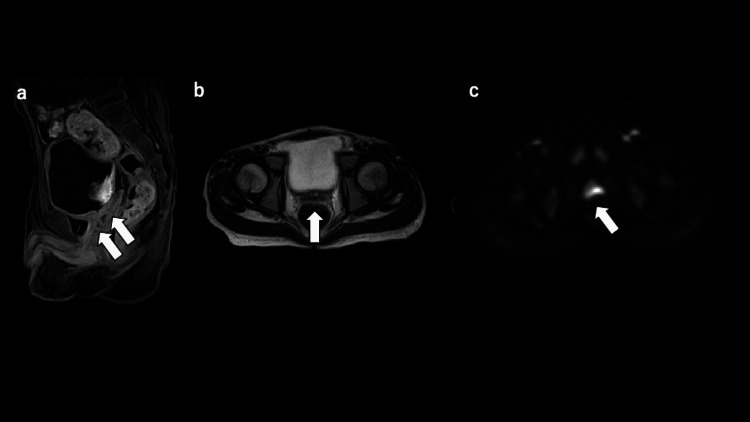
Magnetic resonance imaging. (a) Contrast-enhanced T1-weighted image (sagittal view) revealed peripheral contrast enhancement with internal heterogeneity. (b) The T2-weighted image (axial view) shows a central high-signal and marginal low-signal area. (c) The diffusion-weighted image shows a high signal.

Seven days after his second discharge from the hospital, the patient again developed a fever while receiving oral TFLX therapy and was readmitted to the hospital. Vitamin supplements were continued, and carnitine and zinc supplements were administered. On the fifth day, an MRI scan of the pelvic region was performed to assess the patient’s condition. Contrast-enhanced T1-weighted sequences revealed peripheral contrast enhancement with internal heterogeneity, consistent with multiple abscesses. On T2-weighted MRI, a 10-mm abnormal image with central high-signal and marginal low-signal areas was observed at the head of the prostate gland, which was suspected to be an abscess. In diffusion-weighted images (DWI), the abnormal image also showed a high signal (Figure 2). No congenital abnormalities of the kidney and the urinary tract were revealed by abdominal ultrasound and magnetic resonance urography.

These findings indicated an abscess with inflammatory activity. The intravenous administration of meropenem hydrate (100 mg/kg/day) was initiated for treatment. The MRI test, re-evaluated seven days later, showed improvement. There was no fever after the third discharge, and no relapse has been observed without antibiotic therapy to date. In addition, the patient’s visual impairment gradually recovered after the start of oral vitamin supplementation, and his picky eating tended to improve, resulting in good vitality.

## Discussion

Prostatic abscesses tend to occur in middle-aged men, often secondary to acute prostatitis and are rare in the pediatric population. The most common etiology is gram-negative bacteria, predominantly *Escherichia coli*, which is the leading cause of prostatitis and UTI in men [1]. On the other hand, reports of prostatic abscesses due to *Staphylococcus aureus*, specifically methicillin-resistant *S. aureus* (MRSA), have recently increased [2]. Lee et al. reported a multicenter, retrospective cohort study on acute bacterial prostatitis and abscess formation; according to their report, the cases caused by *P. aeruginosa*, as with this case, accounted for 15% of the total cases [3]. In 2001, Collins et al. reviewed 13 reported cases of prostatic abscesses in newborns. *P. aeruginosa* was the cause in only one case, and 11 out of 13 infant cases required abscess drainage. Of the 13 cases reported during the past 50 years, 10 were published between 1950 and 1960; newer antibiotics, such as third-generation cephalosporins, may allow more frequent treatment of prostatic abscesses without drainage [4]. Only seven cases have been reported in children and adolescents beyond the neonatal period, including this case [5-10] (Table 2). Five of these seven patients were over 10 years of age, and two, including ours, were kindergartners. The pathogens of prostatic abscesses vary: four cases were caused by *S. aureus*, including MRSA, one by *E. coli*, one by *P. aeruginosa*, and one was not detected.

**Table 2:** Prostatic abscess in pediatric patients CT: computed tomography; MRI: magnetic resonance imaging; MRSA: methicillin-resistant *Staphylococcus aureus*; ASD: autism spectrum disorder

Age (years)	Risk factor	Publication year
15	X-linked chronic granulomatous disease	2012 [5]
15	Previous MRSA infection	2012 [6]
11	Pulmonary hypertension	2017 [7]
14	Repetitive forceful influx of contaminated water into the urethra	2019 [8]
6	Poststreptococcal glomerulonephritis	2021 [9]
13	Nil	2021 [10]
5	ASD, limited nutrition	This case

Foster et al. reported the first case of a prostatic abscess due to methicillin-sensitive *S. aureus* in a pediatric patient beyond the neonatal period [7]. Only one patient had a history of post-streptococcal glomerulonephritis and required abscess drainage to improve his symptoms [9]. Ultrasonography was initially conducted in some cases, and all computed tomography or MRI scans were performed to confirm the diagnosis of prostatic abscess.

Our patient’s presentation was unique for he had no known predisposing risk factors, such as immune compromise. However, the patient’s medical history was significant for ASD and biased toward nutrition. His diet was minimal; he only ate polished white rice, “natto,” and sometimes vinegared Japanese seaweed “mozuku” for several months. This restricted diet may have led to vitamin A and C and mineral deficiencies. The patient’s visual impairment was attributed to a vitamin A deficiency.

Children with ASD may sometimes have eating problems, such as food selectivity, nutritional inadequacies, and mealtime behavioral problems [11]. Previous studies reported higher food selectivity and nutrient insufficiency in ASD children than in control children, which were described as insufficient intake of nutrients such as vitamins A, C, B6, B12, D, E, K, and folate and minerals such as phosphorus, zinc, calcium, and iron [11-13].

Previous studies have reported that children with ASD may have an increased risk of bacterial or viral infections [14,15]. Dowd et al. reported that vitamin A supplementation increases cellular immunity in human monocytes or mouse peritoneal macrophages, such as phagocytic and tumoricidal activities. In addition, a significant positive correlation exists between natural killer cell activity and leukocyte vitamin C levels [16]. Adams et al. reported that oral vitamin/mineral supplementation improves the nutritional and metabolic status of children with autism, including improvements in methylation, glutathione, oxidative stress, sulfation, adenosine triphosphate, nicotinamide adenine dinucleotide, and nicotinamide adenine dinucleotide phosphate [17]. There was no recurrence in this case after oral vitamin/mineral supplementation, which indicates that vitamin/mineral deficiency and autism may be considered as the cause of prostatic abscesses.

However, the limitations of this study included the following: 1) Nutrition therapy as vitamin/mineral supplementation at the same time as antibiotic therapy helped avoid recurrent infection. Therefore, we could not ascertain that nutrition therapy alone avoided recurrent infection. 2) We did not study his voiding cystourethrography in the imaging studies. Therefore, the imaging studies for urinary tract abnormalities could not rule out his vesicoureteral reflex. 3) In addition, immunodeficiency workups were performed only for immunoglobulin G, A, and M, and C3 and C4 complement factors. Therefore, we were not able to rule out immunodeficiency in the patient completely.

## Conclusions

We reported a sporadic pediatric case of a five-year-old boy with ASD and various vitamin and mineral deficiencies presumably related to an unbalanced diet and a prostatic abscess caused by *P. aeruginosa*. Nutrition therapy with vitamin/mineral supplementation at the same time as antibiotic therapy helped to avoid recurrent infection.
